# Under the Iceberg

**DOI:** 10.3201/eid3213.AC3213

**Published:** 2026-08

**Authors:** Alexander T. Yu

**Affiliations:** California Department of Public Health, Richmond, California, USA

**Keywords:** wastewater surveillance, art-science connection, Alexander T. Yu, Tip of the Iceberg

**Figure:**
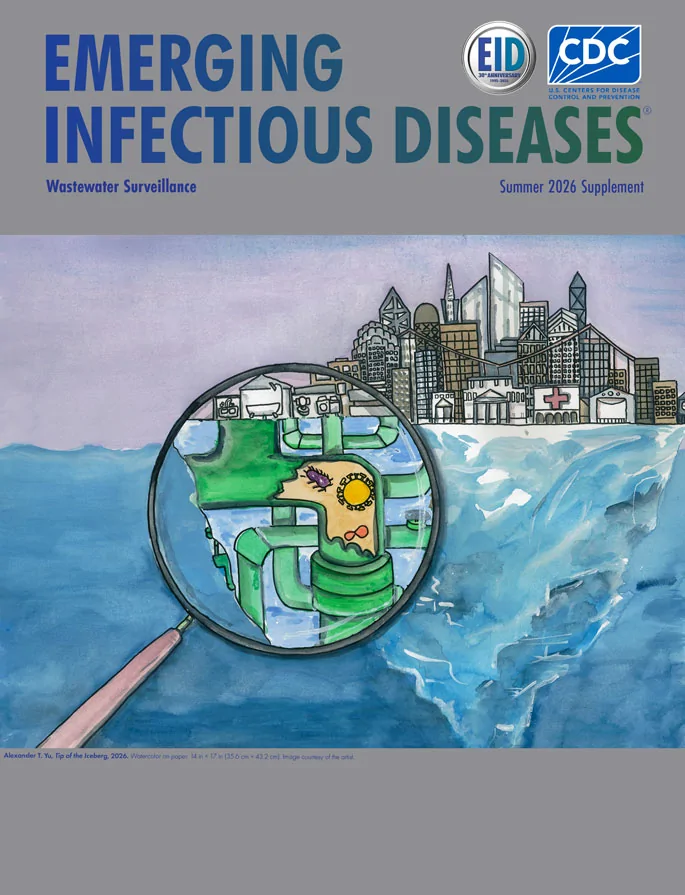
**Alexander T. Yu, *Tip of the Iceberg*, 2026.** Watercolor on paper. 14 in × 17 in. Image courtesy of the artist.

The cover illustration for this supplement to *Emerging Infectious Diseases* depicts a city resting atop an iceberg floating in the ocean. The iceberg is a familiar public health metaphor in infectious disease epidemiology where cases known to traditional public health surveillance systems are often described as the tip of the iceberg—the small visible portion of a much larger burden of disease that remains unseen. Beneath the surface are undiagnosed infections, asymptomatic cases, spontaneous recoveries, unrecorded deaths, and other uncounted incidences due to barriers to healthcare access, limitations of testing, and a multitude of other reasons.

As a public health medical epidemiologist and infectious diseases physician, I have spent the past decade trying to illuminate what lies underneath the tip of the iceberg. Wastewater surveillance (WS), the focus of this supplement issue, has been an incredible tool, allowing public health to do just that. WS works by collecting samples from sewage, which everyone living in sewered communities contributes to, and testing it for the presence and quantity of different pathogens. As such, WS can capture information from entire communities, regardless of symptoms or healthcare-seeking behavior. That process contrasts with traditional surveillance systems, which rely on test results from individual persons. WS can provide early warning of outbreaks, identify emerging pathogens, monitor disease trends, and complement traditional clinical surveillance. This form of surveillance can also generate meaningless, difficult to interpret, or inactionable data. As the illustration depicts, the sewershed is large and diverse. Interpreters of the data the system generates may not be able to readily discern the source(s) of salient findings, be they from homes, hospitals, or farms, or even whether they originate from humans or animals.

WS is exciting but still relatively novel, unheard of by many, including those in healthcare and public health. There remains the basic need to understand how to translate noisy wastewater data that is meaningless by itself into a clear message interpretable to all (e.g., public health, health care providers, the public). Before I began a career in medicine and public health, I worked as a freelance cartoonist and illustrator for a variety of newspapers. Now, as a public health practitioner often trying to communicate about public health and WS findings, I have found myself leaning on lessons learned from that time as a cartoonist and illustrator. An essential element of cartooning and illustration is to convey simple and clear messages or stories that can be interpreted similarly by many different viewers and still carry nuance and complexity.

To capture this moment in the WS field and the essence of what this supplement hopes to improve, I wanted to create an illustration that could communicate simply what WS is and the role it can play in public health by leaning on the familiar iceberg metaphor. In the illustration, a magnifying glass focused under the iceberg reveals a network of wastewater pipes and the microorganisms flowing through them. This hidden infrastructure carries traces of entire communities, creating an opportunity to observe population health from a different perspective. By revealing part of the iceberg that has long remained unseen beneath the surface, WS offers a new perspective on an old challenge: understanding the health of populations through signals that are often hidden from view.

